# Genetics and precision health: the ecological fallacy and artificial intelligence solutions

**DOI:** 10.1186/s13040-023-00327-z

**Published:** 2023-03-13

**Authors:** Scott M. Williams, Jason H. Moore

**Affiliations:** 1 Departments of Population and Quantitative Health Sciences, Department of Genetics and Genome Sciences, and Cleveland Institute for Computational Biology, Case Western Reserve University School of Medicine, Cleveland, OH USA; 2 Department of Computational Biomedicine, Cedars-Sinai Medical Center, Los Angeles, CA USA

Human genetics began as a field that used family-based linkage studies to identify genes associated with specific phenotypes. This approach was highly successful in finding genes of high penetrance that were essentially Mendelian in their inheritance patterns and highly predictive of phenotypes. This approach truly identified individuals at risk of disease within families based usually on a single mutation and in current lingo could provide precision health. Take for example, infants born with phenylketonuria (PKU) resulting from mutations in a single gene, phenylalanine hydroxylase. These children can be prevented from having adverse outcomes by a simple elimination of phenylalanine from the diet. This is an excellent example of precision health, where a serious outcome can be avoided or at least minimized by a targeted intervention based on an individual’s genotype.

Over the last 30 years the study design of choice for human geneticists moved from linkage to genetic epidemiological using increasingly large case–control association studies [1]. This approach has succeeded in identifying many genes that associate with disease, and these studies have been extremely informative in dissecting the underlying biological bases of many diseases. Few of these genes on their own are, however, highly predictive of disease. Nonetheless, genetics is still presented as a means to provide precision medicine for both targeted treatment and prevention. There is little doubt that the potential of genetics in these realms exists, but it is important to evaluate the limitations of research as currently performed under the guise of precision medicine, and how we can bring the field closer to these aspirations. Specifically, in the current large case control studies of complex genetic disease, variants and/or genes are identified that increase or decrease disease risk. It is important to note that the estimates of risk presented are in fact the average effects across the study samples (populations) [2] and not ones representing individual risk as often is the case in highly penetrant Mendelian disorders. Take for example a specific case where a single mutation has been strongly associated with Alzheimer’s disease (AD). The *ApoE4* allele was discovered 30 years ago and still represents the single most important genetic risk factor for this disease where it accounts for as much as ~ 25% of the heritability of disease liability and as much as 20% of the attributable risk in Europeans [3, 4]. This allele associates with AD in virtually every study across multiple populations studied. However, the odds conferred by this allele is highly variable and population specific. In African Americans, the odds are estimated to be ~ 5 in individuals homozygous for *ApoE4* whereas in Japanese the same genotype has an OR of ~ 30 [5, 6]. Based on these findings alone we should be wary of the ability of association analyses to provide individual level risk. For example, what is risk in a person of multiple ancestries?

More recently human genetics has adapted the use of polygenic risk scores, which, although calculated many ways, are compilations of risk as estimated by many variants assessed simultaneously [7–9]. At issue is whether the genetic factors derived in this way can be applied to the study of individual risk as they are often purported to do. The first question that needs to be addressed is whether the estimate of risk, presented as effect sizes or odds ratios, can be translated from population level results where they represent the average effect of alleles to individuals who do or do not carry them. The tenuous leap from population average odds to individual risk has been described in the epidemiological literature as the “ecological fallacy” or as the inference derived from group analyses to individuals [10] and has been noted with respect to PRSs explicitly [7, 11]. Effects sizes derived from genetic epidemiological studies by their very nature suffer from this and PRSs are simply the compilation of multiple ecological fallacies tallied that carry a substantial amount of uncertainty [12]. And as noted above for *ApoE*, the effect sizes and hence predictability do not translate well across diverse populations. Neither do the PRSs themselves [13–15]. What causes variation in prediction accuracy and underlying pathoetiology is key to precision medicine but cannot be resolved by population level estimates. That said, we do recognize that in many cases PRSs at the extreme can be predictive of individual risk [16], but for the vast majority of people and for those of different ancestries they suffer from both poor transferability and the ecological fallacy.

One approach to address the ecological fallacy is to attempt to identify subgroups of subjects more representative of each individual’s risk. This requires large sample sizes and powerful computational and statistical methods capable of identifying subgroups defined by different combinations of genetic and non-genetic factors. The types of genetic effects that can define subgroups not represented by population-level estimates include non-additive gene–gene and gene-environment interactions and genetic heterogeneity. Artificial intelligence methods including machine learning are ideally suited to risk prediction when the patterns to be detected and modeled are non-additive and/or heterogeneous in nature. An example of a machine learning method designed specifically for detecting subgroups defined by these kinds of genetic effects is learning classifier systems or LCS [17]. What makes an LCS different from other statistical and computational methods is that it optimizes a set of rules relating genetic measures to phenotype where each rule defines a subgroup. The collection of rules is evaluated as the model. This is different from most other analytical methods that develop one model on the entire sample. Methods such as LCS have the potential to alleviate some of the problems associated with the ecological fallacy by generating risk estimates that are much more closely aligned to each individual than those derived from an entire sample representing a heterogenous population. Integrating machine learning models with PRSs will be necessary to derive better risk estimates for individuals. This will facilitate precision medicine informed by big data from observational studies.
